# Cognitive domain-specific impairments and associated risk factors in type 2 diabetes mellitus: a cross-sectional observational study based on neuropsychological assessment from Xiamen, China

**DOI:** 10.7717/peerj.20292

**Published:** 2025-11-04

**Authors:** Xueling Xiao, Luling Chen, Jingqi Liu, Jiayan Cai, Manxiang Deng

**Affiliations:** Department of Geriatrics, Zhongshan Hospital Xiamen University, Xiamen, Fujian, China

**Keywords:** T2DM, Cognitive impairment, Cognitive domain, Influencing factor

## Abstract

**Background:**

Type 2 diabetes mellitus (T2DM) is associated with an increased risk of cognitive impairment, yet limited research has been conducted in subtropical regions of China.

**Objective:**

To examine the characteristics of cognitive impairment and identify the potential risk factors in patients with T2DM in Xiamen.

**Methods:**

This cross-sectional observational study included 84 patients with T2DM from Zhongshan Hospital Xiamen University. Patients were grouped based on their Montreal Cognitive Assessment (MoCA) scores into a cognitively impaired group (T2DM-CI group, *n* = 52) and a cognitively normal group (T2DM-NCI group, *n* = 32). Multivariate logistic regression was used to identify independent risk factors.

**Results:**

Among the 52 patients in the T2DM-CI group, the most commonly affected cognitive domains were executive function (82.7%), language (75.0%), memory (61.5%), and attention (48.1%), with 59.6% exhibiting impairments in three or more domains. Compared with the T2DM-NCI group, the T2DM-CI group showed poorer performance in most MoCA subdomains—including visuospatial/executive function, language, delayed recall, abstraction, and orientation—as well as in individual cognitive domain tests (all *P* < 0.05), except for the Clock Drawing Test. Older age (OR = 1.167, 95% CI [1.045–1.303], *P* = 0.006) and higher lipoprotein (a) levels (OR = 1.109, 95% CI [1.020–1.205], *P* = 0.015) were independently associated with cognitive impairment in T2DM patients.

**Conclusion:**

Cognitive impairment in T2DM affects multiple domains, with executive dysfunction most prominent. Age and elevated lipoprotein(a) may increase risk. Routine cognitive screening is warranted, particularly in older patients and those with vascular risk factors.

## Introduction

Type 2 diabetes mellitus (T2DM) and dementia are both age-related chronic conditions that have become significant public health challenges worldwide. Approximately 9% of the global population suffers from diabetes, with T2DM accounting for over 90% of cases (Zheng, Ley & Hu, 2018). A substantial body of evidence indicates a strong association between T2DM and cognitive impairment, with around 60–70% of patients experiencing varying degrees of cognitive decline (You et al., 2021; Jia et al., 2020; Jayaraj, Azimullah & Beiram, 2020). T2DM is not only a major risk factor for vascular dementia but is also closely linked to an increased risk of non-vascular dementia, such as Alzheimer’s disease, with affected individuals exhibiting a 1.5 to 2.5-fold higher risk compared to those without diabetes (Srikanth et al., 2020). Furthermore, T2DM also heightens the risk of cognitive impairment progressing to dementia (Maimaitituerxun et al., 2023). The underlying mechanisms are multifactorial (Rizzo et al., 2022; Anita et al., 2022), involving vascular damage, chronic hyperglycemia, insulin resistance, oxidative stress, amyloid-beta accumulation, tau hyperphosphorylation, and decreased levels of neurotrophic factors. Although the pathological basis and epidemiological features of T2DM-related cognitive impairment have received some attention, it remains underrecognized in clinical practice, and effective preventive and therapeutic strategies are lacking. Early identification and management are therefore critically important.

Currently, there is no unified classification standard for the clinical manifestations of T2DM-related cognitive impairment, and the associated risk factors remain inconclusive. While the relationship between T2DM and cognitive dysfunction has been widely studied, data specific to subtropical regions of China remain limited. Considering potential differences in environmental factors, lifestyle, and population characteristics in these areas, it is necessary to conduct localized research to provide regionally representative clinical data and to support early screening and individualized intervention strategies.

To address this gap, we conducted a study in Xiamen, a subtropical coastal city in southeastern China, to systematically evaluate cognitive function and its influencing factors in patients with T2DM. A comprehensive battery of cognitive assessments was employed, including the Mini-Mental State Examination (MMSE), Auditory Verbal Learning Test (AVLT), Digit Span Forward Test (DST), Verbal Fluency Test (VFT), Digit Symbol Substitution Test (DSST), Stroop Color and Word Test (SCWT), Block Design Test (BD), and Clock Drawing Test (CDT), covering multiple cognitive domains. This study aims to characterize the patterns of cognitive impairment in this specific regional population and identify potential risk factors based on demographic and clinical characteristics. The findings provide regionally relevant evidence to complement existing literature on cognitive dysfunction in individuals with T2DM.

## Materials & Methods

### Research design and populations

This cross-sectional observational study included hospitalized patients aged 46–82 years diagnosed with T2DM at Zhongshan Hospital Xiamen University between March 2022 and June 2023. T2DM was diagnosed according to the criteria of the American Diabetes Association (ADA). Patients were admitted for various internal medicine-related conditions such as poor glycemic control, diabetes-related complications (*e.g.*, nephropathy, neuropathy), or comorbid cardiovascular and metabolic diseases.

Exclusion criteria included: (1) schizophrenia or intellectual disability; (2) severe depressive disorder; (3) neurological conditions such as stroke, dementia with Lewy bodies, Parkinson’s disease, neurosyphilis, hydrocephalus, brain tumors, epilepsy, or encephalitis; (4) vitamin deficiency or thyroid dysfunction; (5) cardiac, pulmonary, hepatic, or renal insufficiency; (6) history of chronic alcohol or substance abuse; (7) acute diabetic complications including ketoacidosis or hyperosmolar hyperglycemic state; (8) inability to cooperate with cognitive assessments; and (9) incomplete clinical or cognitive data.

This study was approved by the Ethics Committee of Zhongshan Hospital, Xiamen University (2023-112), and conducted in accordance with the Declaration of Helsinki. The requirement for informed consent was waived due to the retrospective nature of the study. All patient data were anonymized, and confidentiality was strictly maintained throughout the research process.

### Clinical data collection

The study recorded participants’ baseline characteristics, including sex, age, and years of education, as well as the following clinical data potentially related to cognitive function: duration of diabetes; history of smoking and alcohol consumption; occurrence of more than three hypoglycemic episodes (defined as symptomatic episodes with self-monitored blood glucose <3.9 mmol/L or requiring assistance); presence of diabetic complications, including diabetic retinopathy (diagnosed by ophthalmologists through fundus photography and related assessments), diabetic peripheral neuropathy (assessed using clinical symptoms, physical examination, and nerve conduction studies when available), and diabetic nephropathy (defined as a urinary albumin-to-creatinine ratio ≥ 30 mg/g or estimated glomerular filtration rate (eGFR) < 60 mL/min/1.73 m^2^ for at least 3 months); use of metformin and insulin (included as binary variables: yes or no); comorbid hypertension and coronary heart disease; and dyslipidemia status (defined according to the Chinese Guidelines for the Prevention and Treatment of Dyslipidemia in Adults: total cholesterol ≥ 6.2 mmol/L, LDL-C ≥ 4.1 mmol/L, HDL-C < 1.0 mmol/L, or triglycerides ≥ 2.3 mmol/L). Laboratory indicators included body mass index (BMI), glycated hemoglobin (HbA1c), fasting blood glucose, lipoprotein(a)—measured from a single fasting venous blood sample—serum uric acid, and homocysteine levels. All variables were included in the analysis due to their potential associations with cognitive performance in individuals with T2DM. No missing data were present in the final dataset used for analysis.

### Cognitive performance evaluation

All cognitive assessments were conducted by physicians and nurses in our department who received standardized training and were blinded to patients’ clinical and grouping information to minimize observer bias. Global cognitive function was assessed using the Mini-Mental State Examination (MMSE), Montreal Cognitive Assessment (MoCA), Global Deterioration Scale (GDS), and Clinical Dementia Rating (CDR) scales. Normal cognition was defined as meeting all of the following criteria simultaneously: MMSE ≥ 27, MoCA ≥ 26, GDS = 1, and CDR = 0. Cognitive impairment was diagnosed when either (1) MoCA <26, CDR ≥ 0.5, and GDS ≥ 2, or (2) MMSE < 27, CDR ≥ 0.5, and GDS ≥ 2.

Domain-specific cognitive abilities were assessed as follows: memory using the Auditory Verbal Learning Test (AVLT); language fluency using the Verbal Fluency Test (VFT); executive function using the Digit Symbol Substitution Test (DSST) and Stroop Color-Word Test (SCWT); attention using the Digit Span forward Test (DST); visuospatial ability using the Clock Drawing Test (CDT) and Block Design (BD); and activities of daily living using the ADL scale.

Domain-specific cognitive impairments were defined according to previously published studies and relevant clinical guidelines (Gong, 1992; Han, 2018; Gu et al., 2009; Lafont et al., 2010). The cut-off values were set as follows: Memory (AVLT N5): ≤5 for ages 50–59, ≤4 for ages 60–69, and ≤3 for ages 70–80; Language (VFT/AFT-A): ≤12 for junior high school education, ≤13 for high school, and ≤14 for college; Executive function (DSST): <29; Attention (DST): ≤5 for illiterate individuals, ≤6 for those with primary education, and ≤7 for middle school education and above; Visuospatial ability (BD): ≤10 for illiterate individuals, ≤15 for primary education, and ≤20 for middle school education and above.

### Statistical analyses

Data analyses were conducted using SPSS 26.0 statistical software (IBM Corp., Armonk, NY, USA). Continuous variables were presented as mean ± standard deviation or median (25–75th percentile) for normal or non-normal distributions, respectively. Group comparisons for normally distributed measurement data were performed using the independent samples *t*-test, while comparisons for non-normally distributed data were performed using the Mann–Whitney U test. Percentage representation was used for count data, with chi-square tests employed for group comparisons. All statistical tests were two-sided, and a *P*-value of less than 0.05 was considered statistically significant. To control for false positives in multiple comparisons, the false discovery rate (FDR) correction was applied to comparisons across multiple cognitive domains.

## Results

### Cognitive impairment in patients with T2DM

A total of 84 patients with T2DM were included in this study and divided into two groups: the T2DM-NCI group (*n* = 32) without cognitive impairment and the T2DM-CI group (*n* = 52) with cognitive impairment. The participants’ ages ranged from 46 to 82 years, with a mean of 64.73 ± 8.46 years. Females accounted for 44.0% and males 56.0% of the cohort. Diabetes duration was less than 10 years in 45.2% of patients, 10–19 years in 35.7%, and ≥20 years in 19.0%. Smoking and alcohol use were reported by 32.1% and 29.8% of patients, respectively. Educational levels varied, with most patients having 1–12 years of education. Full sociodemographic details are shown in Table 1.

**Table 1:** Sociodemographic characteristics of patients with type 2 diabetes mellitus.

Sociodemographic characteristics	n	Percentage (%)
Gender		
Female	37	44.0
Male	47	56.0
Age (year)		
46∼59	21	25.0
60∼69	37	44.0
70∼82	26	31.0
Smoking (yes)	27	32.1
Drinking (yes)	25	29.8
Duration of diabetes (years)		
<10	38	45.2
10∼19	30	35.7
≥20	16	19.0
Years of education		
0	3	3.6
1∼6	25	29.8
7∼9	24	28.6
9∼12	19	22.6
>12	13	15.4

As shown in Table 2, after controlling for multiple comparisons using the FDR method, the T2DM-CI group scored significantly lower than the T2DM-NCI group in most MoCA sub-items (except for naming and attention sub-items), and the majority of individual cognitive domain tests, including executive function, memory, language, and visuospatial abilities (adjusted *P* < 0.05). No significant differences were found in the MoCA naming sub-item, attention sub-items, or the CDT after FDR correction.

**Table 2:** Cognitive test scores in the T2DM-NCI and T2DM-CI groups with FDR-adjusted *P* values.

Individual cognitive domain	T2DM-NCI (*n*= 32)	T2DM-CI (*n*= 52)	*Z* value	*P* value	FDR-adjusted *P* value
MoCA scores	27 (26, 27.75)	22 (19, 23)	−7.702	<0.001	
MMSE scores	29 (29, 30)	26 (24, 27)	−6.544	<0.001	
MOCA test					
Visuospatial/executive	4.5 (4, 5)	3 (2, 4)	−4.104	<0.001	0.007
Language	3 (2, 3)	1 (1, 2)	−5.588	<0.001	0.007
Naming	3 (3, 3)	3 (3, 3)	−0.784	0.433	0.433
Abstraction	1 (1, 2)	1 (0, 1)	−4.287	<0.001	0.007
Attention	6 (6, 6)	6 (5, 6)	−1.875	0.061	0.071
Orientation	6 (6, 6)	6 (5, 6)	−4.087	<0.001	0.007
Memory delayed recall	4 (3, 4)	1 (0, 2)	−6.666	<0.001	0.007
Memory					
AVLT short time	6 (4, 8)	4 (2, 6)	−3.717	<0.001	0.011
AVLT long time	5.5 (4, 7)	3 (1, 5)	−3.830	<0.001	0.011
Language					
VFT executive	15.5 (12, 18)	11 (8, 13)	−3.821	<0.001	0.011
DSST	34.50 (30.25, 39.5)	20 (16, 30.2)	−6.769	<0.001	0.011
SCWT-A(s)	13.08 (10.55, 15.85)	18.36 (13.71, 24.15)	−3.455	0.001	0.011
SCWT-B(s)	19.73 (17.05, 24.23)	24.26 (19.53, 35.29)	−2.552	0.011	0.015
SCWT-C(s)	45.20 (37.35, 60.28)	60.46 (47.43, 80.18)	−3.114	0.002	0.011
SCWT C-A(s)	32.44 (25.08, 45.75)	43.24 (33.45, 56.03)	−2.496	0.013	0.016
Attention					
DST	8 (8, 10)	7 (5, 9)	−3.212	0.001	0.011
Visuospatial					
BD	32 (26, 33.5)	24 (20, 28.75)	−4.504	<0.001	0.011
CDT	10 (7, 10)	9 (7, 10)	−1.051	0.293	0.322

As detailed in Table 3, the impairment of cognitive domains in the T2DM-CI group (*n* = 52) varied across memory, attention, executive function, visuospatial skills, and language. The primary affected cognitive domains were executive function (43 cases, 82.7%), language (39 cases, 75%), memory (32 cases, 61.5%), and attention (25 cases, 48.1%), with amnestic cognitive impairment accounting for 61.5% (32 cases). Among the 52 T2DM-CI patients, only 15.4% showed impairment in a single cognitive domain, while 59.6% demonstrated impairment in three or more domains.

**Table 3:** Distribution of impaired cognitive domains and number of affected domains among T2DM patients with cognitive impairment.

Cognitive domain	n (%)		Number of impaired domains	n (%)
Memory	32 (61.5%)		1	8 (15.4%)
Language	39 (75.0%)		2	13 (25.0%)
Executive function	43 (82.7%)		3	14 (26.9%)
Attention	25 (48.1%)		4	10 (19.2%)
Visuospatial ability	12 (23.1%)		5	7 (13.5%)
			≥3	31 (59.6%)

### Potential risk factors for cognitive impairment in T2DM

As shown in Table 4, the clinical characteristics of diabetic patients in the T2DM-CI and T2DM-NCI groups are presented. By comparing the two groups, the study explored potential risk factors associated with cognitive impairment in patients with T2DM. Univariate regression analysis indicated that age, diabetic retinopathy, occurrence of more than three hypoglycemic episodes, and lipoprotein (a) levels were statistically associated with cognitive dysfunction (*P* < 0.05). Variables with a *P*-value of less than 0.1 in the univariate analysis—namely age, years of education, duration of diabetes, presence of diabetic retinopathy, presence of diabetic peripheral neuropathy, lipoprotein (a) levels, more than three episodes of hypoglycemia, and history of metformin medication—were included in the multivariate logistic regression model. This model identified age (OR = 1.167, 95% CI [1.045–1.303], *P* = 0.006) and lipoprotein (a) levels (OR = 1.109, 95% CI [1.020–1.205], *P* = 0.015) as factors independently associated with cognitive impairment in T2DM patients.

**Table 4:** Clinical characteristics of patients in the T2DM-CI group and T2DM-NCI group.

	T2DM-NCI (*n*= 32)	T2DM-CI (*n*= 52)	*Z*/*t*/*χ*^2^	*P*
Age (years)	60.75 ± 8.22	67.17 ± 7.72	−3.615	0.001
Female (%)	50.0	40.4	0.743	0.389
Education (year)	9 (6.5, 12)	9 (5, 12)	−1.827	0.068
Smoking (%)	37.5	28.8	0.680	0.410
Drinking (%)	34.4	26.9	0.526	0.468
Duration of diabetes (years)	9 (5, 12.5)	12 (7.25, 16)	−1.884	0.06
Hypoglycemia episodes > 3 (%)	18.8	53.8	10.128	0.001
Metformin (%)	75	55.8	3.146	0.076
Insulin (%)	28.1	42.3	1.711	0.191
Microvascular complication				
Peripheral neuropathy (%)	68.8	84.6	2.962	0.085
Diabetic nephropathy (%)	31.3	32.7	0.019	0.891
Retinopathy (%)	3.1	42.3	15.295	0.000
HbA1c(%)	7.8 (6.5, 8.9)	7.7 (6.7, 8.7)	−0.396	0.692
Fasting blood glucose (umol/L)	8.2 ± 2.3	8.0 ± 3.4	0.295	0.769
BMI (kg/m^2^)	23.55 (22.23, 26.08)	23.78 (21.92, 25.24)	−0.585	0.559
Dyslipidemia (%)	71.9	84.6	1.992	0.158
Hypertension (%)	53.1	67.3	1.690	0.194
Homocysteine (umol/L)	9.7 (8.4, 13.3)	10.4 (9.0, 14.2)	−0.898	0.369
Blood uric acid (umol/L)	348 (282, 434)	315 (258, 404)	−1.349	0.177
Lipoprotein (a) (mg/dL)	5.8 (3.2, 13.2)	30.8 (10.6, 73.1)	−5.205	<0.001

## Discussion

### Clinical characteristics of cognitive impairment associated with T2DM

In this study, 61.9% of patients with T2DM exhibited cognitive impairment, a prevalence consistent with findings from both domestic and international studies (You et al., 2021; Jia et al., 2020; Jayaraj, Azimullah & Beiram, 2020; Dao, Choi & Freeby, 2023). This similarity suggests that the risk of diabetes-related cognitive impairment may be comparable across different populations, regardless of ethnic background. Patients in the T2DM-CI group had significantly lower total scores on the MoCA and MMSE tests, suggesting a general decline in cognitive function across multiple domains. This finding is consistent with previous research. Furthermore, 52 patients were identified as having cognitive impairment using MoCA criteria, compared to only 28 identified using MMSE, indicating that the MMSE may have lower sensitivity in detecting cognitive impairment in individuals with T2DM. This discrepancy may be partly explained by the MMSE’s limited assessment of certain cognitive domains, such as language and executive function. Consequently, it may underestimate impairment in these areas. In addition, MMSE performance can be influenced by educational background, with individuals who have higher education levels or greater cognitive reserve potentially achieving better scores despite underlying deficits.

Patients with T2DM commonly exhibit cognitive impairment in various domains including prefrontal executive function, memory, information processing speed, and attention (Whitelock et al., 2021; Dyer et al., 2021; Xie et al., 2022). Our study revealed deficits in memory, attention, executive function/information processing speed, language, and visuospatial abilities, with visuospatial impairment being comparatively less prominent. Within the cognitive impaired group, a significant portion exhibited amnestic cognitive impairment (61.5%) and multidomain cognitive dysfunction (59.6%). Executive dysfunction was particularly prevalent (82.7%), consistent with previous research findings (Palta et al., 2014; Chen et al., 2023; Ryan, Van Duinkerken & Rosano, 2016). Executive dysfunction in diabetic patients may impair self-management and treatment adherence, warranting clinical attention. Mild amnestic cognitive impairment is recognized as a prodromal stage of AD, with a markedly increased risk of progression to AD compared to the general elderly population (Lee et al., 2014). Neuropathological changes in AD predominantly involve regions such as the temporoparietal-occipital junction, hippocampus, medial temporal lobe, and amygdala. These regions are crucial for memory processes. As a result, patients with AD commonly present with significant memory impairments, especially in episodic memory. Delayed recall is more severely affected than in vascular dementia. The high prevalence of amnestic cognitive impairment observed in our study may suggest a link between T2DM and memory dysfunction characteristic of AD, potentially exacerbating neurodegenerative processes.

However, non-amnestic impairments—in particular, executive function, attention, processing speed, and language—were more frequently observed in T2DM patients with cognitive impairment. Furthermore, most cognitively impaired patients exhibited deficits across multiple domains, suggesting that both neurodegenerative and vascular mechanisms may contribute to the cognitive profile seen in T2DM. Previous studies have shown that the neural pathway involving the frontal lobe, striatum, globus pallidus, thalamus, and cortex is particularly susceptible to ischemic damage. This pathway plays a central role in regulating executive function, information processing speed, attention, and emotion (Kalaria, 2018). Impairments in executive function may hinder patients’ ability to plan, organize, and adhere to complex diabetes self-care behaviors, including medication management, blood glucose monitoring, and lifestyle modifications. Patients with vascular dementia commonly exhibit widespread white matter lesions such as leukoaraiosis, lacunar infarction, and varying degrees of brain atrophy. These lesions can disrupt neural circuits connecting key areas of the frontal cortex and lead to impaired executive functions (Inoue et al., 2023; Hu et al., 2021). Compared to AD, patients with vascular dementia more often exhibit impairments in executive function and attention. The coexistence of AD-like and vascular cognitive features in patients with T2DM suggests that beyond neurodegenerative processes, vascular factors also contribute to the development of cognitive impairment. T2DM-related cognitive impairment appears to reflect a mixed pathology, with vascular mechanisms potentially playing a predominant role. Further studies incorporating neuroimaging and other modalities are warranted to elucidate the underlying brain changes in this population.

### Risk factors for cognitive impairment among patients with T2DM

Multiple studies have consistently demonstrated that age is a major risk factor for dementia—including AD, vascular dementia, and other neurodegenerative conditions—and is inversely associated with cognitive function. The incidence of cognitive decline increases exponentially with age, approximately doubling every five years. This is likely due to the accumulation of vascular and neurodegenerative changes in the aging brain (Institute for Health Metrics and Evaluation, 2020; Yuan et al., 2022). Elderly individuals with T2DM face an even higher risk, with a 1.5- to 2-fold increased likelihood of developing cognitive impairment compared to non-diabetic peers (Cukierman, Gerstein & Williamson, 2005; Reuter-Lorenz & Cooke, 2016). This heightened vulnerability may be attributed to the higher prevalence of macrovascular and microvascular complications, brain atrophy, and lacunar infarctions. Additional factors include age-related changes such as white matter abnormalities, reduced brain volume, vascular dysfunction, oxidative stress, and accumulation of advanced glycation end products. In the present study, age was identified as an independent predictor of cognitive impairment in T2DM (OR = 1.167), albeit slightly lower than previously reported estimates. These findings suggest that age may play an important role in the development of cognitive decline in patients with T2DM and support the need for early screening and intervention in older diabetic populations.

In this study, lipoprotein (a) was identified as an independent risk factor for cognitive impairment in patients with T2DM (OR = 1.109), a relatively uncommon and under-investigated finding. Lipoprotein (a) shares structural similarities with low-density lipoprotein (LDL) and is closely related to plasminogen, with its levels primarily determined by genetic factors. Its physiological function involves inhibition of fibrinolysis. However, excessive accumulation of lipoprotein (a) within vascular walls can accelerate the development of atherosclerosis. This may contribute to microvascular damage and increase the risk of cardiovascular and cerebrovascular diseases. Prior studies have linked elevated lipoprotein (a) levels with an increased risk of stroke and coronary heart disease (Mehta et al., 2022; Li et al., 2022). The potential mechanism underlying its impact on cognitive function may involve vascular injury and impaired cerebral perfusion due to arteriosclerotic changes. Although direct evidence connecting lipoprotein (a) with cognitive impairment in individuals with T2DM remains limited, the present findings suggest that lipoprotein (a) may serve as a promising biomarker for early identification of cognitive decline in this population. Given the relatively limited literature on this topic, especially within diabetic cohorts, future studies employing prospective designs and involving larger, diverse populations are warranted to validate these findings and explore their implications for risk stratification and targeted intervention strategies.

Considering the associations with age and lipoprotein (a), routine cognitive screening may help identify high-risk T2DM patients and facilitate early, targeted interventions. Early identification of cognitive impairment could allow timely intervention, including optimization of glycemic control, management of vascular risk factors, and lifestyle modifications to slow cognitive decline. Although direct treatments targeting lipoprotein (a) are limited, monitoring its levels might help stratify patients at higher risk, guiding personalized prevention strategies. Emerging therapies aimed at reducing lipoprotein (a) and associated vascular damage warrant further investigation for their potential to preserve cognitive function in this population.

Despite its contributions, this study has several limitations. First, the relatively small sample size limited the statistical power and the number of risk factors that could be comprehensively analyzed. This may partly explain the lack of a significant association between blood pressure and cognitive impairment, despite prior evidence supporting this link. Second, the assessment of glycemic control was limited to HbA1c levels, without further classification of control status. This may have overlooked the broader impact of glucose variability. Third, the study population consisted primarily of hospitalized patients. Some of these patients may have experienced recent fluctuations in blood glucose levels or psychological stress, potentially influencing cognitive test performance and introducing selection bias. This focus on hospitalized individuals may limit the generalizability of our findings to the broader population of patients with T2DM. Additionally, as a cross-sectional observational study, this research lacked neuroimaging or biomarker data (*e.g.*, brain MRI or cerebrospinal fluid measures). Such data are valuable for the precise characterization of cognitive impairment. Furthermore, causal relationships cannot be inferred due to the study design. The study also did not account for the use of centrally acting medications such as benzodiazepines due to incomplete medication records. Future large-scale, prospective studies are needed to validate these findings. Such studies should better assess the role of glycemic and blood pressure control, explore underlying mechanisms, and improve risk stratification and prevention strategies for cognitive decline in patients with T2DM.

## Conclusions

In conclusion, this study provides regionally specific evidence from the subtropical areas of China that patients with T2DM and cognitive impairment exhibit varying degrees of overall cognitive decline as well as deficits in specific cognitive domains. Executive function is particularly affected, with language, memory, and attention also being major affected cognitive domains. Multiple cognitive domains are often simultaneously impaired. MoCA is more sensitive than MMSE in detecting cognitive impairment in T2DM. It is important to use domain-specific cognitive tests for comprehensive evaluation. Tailored cognitive rehabilitation should be guided by the specific patterns of impairment observed. Additionally, for older T2DM patients with elevated lipoprotein (a) levels, routine cognitive screening and timely protective interventions are critical to prevent or slow cognitive decline.

## Supplemental Information

10.7717/peerj.20292/supp-1 Supplemental Information 1 Raw data

## Additional Information and Declarations

### Data Availability

The following information was supplied regarding data availability: The anonymised dataset is available in the Supplementary File.
